# Assessing the inhibitory properties of a latent inhibitor in flavor-aversion learning

**DOI:** 10.3758/s13420-021-00490-5

**Published:** 2021-10-19

**Authors:** Unai Liberal, Gabriel Rodríguez, Geoffrey Hall

**Affiliations:** 1 University of the Basque Country (UPV/EHU), Donostia/San Sebastián, Spain; 2 University of York, York, UK; 3 University of New South Wales, Sydney, Australia

**Keywords:** Latent inhibition, Inhibitory learning, Flavor aversion, Rats

## Abstract

In Experiment 1, rats received 16 nonreinforced trials of exposure to a flavor (A) that was subsequently used as the conditioned stimulus in flavor-aversion conditioning. In the critical condition, Flavor A was presented in compound with a different novel flavor on each of the eight daily trials. This treatment produced latent inhibition, in that this preexposure retarded conditioning just as did 16 trials with A alone. Rats in the control conditions, given no preexposure or exposure just to the sequence of novel flavors, learned readily. Experiment 2 examined the effects of these forms of preexposure on performance on a summation test, in which Flavor A was presented in compound with a separately conditioned flavor (X). The preexposure procedure in which A was presented along with novel flavors rendered A effective in inhibiting the response conditioned to X on that test. The conclusion, that this form of training can establish the target stimulus as a conditioned inhibitor, is predicted by the account of latent inhibition put forward by Hall and Rodríguez (2010) which proposes that the latent inhibition effect is a consequence both of a reduction in the associability of the stimulus and of a process of inhibitory associative learning that opposes the initial expectation that a novel event will be followed by some consequence.

When Lubow and Moore (1959) first demonstrated that initial nonreinforced exposure to the event to be used as a conditioned stimulus (CS) retards the acquisition of the conditioned response (CR), they labeled the phenomenon *latent inhibition.* The use of the term *inhibition* to refer to this phenomenon served to stress that subsequent learning was retarded (rather than being facilitated, as had been observed in some other preexposure procedures). It soon became clear that such a preexposed stimulus did not possess all the properties expected of a Pavlovian conditioned inhibitor (e.g., Rescorla, 1969). First, it was found that nonreinforced preexposure retards subsequent inhibitory learning rather than facilitating it, as it would be expected if such a preexposure endowed the stimulus with inhibitory properties of the sort governed by a stimulus explicitly trained as a conditioned inhibitor (e.g., Wagner & Rescorla, 1972). Second, it was found that a stimulus preexposed in the absence of reinforcement does not reliably “pass” a summation test—that is, it does not show a special capacity for suppressing the ability of a concurrently presented excitatory CS to evoke its CR (e.g., Reiss & Wagner, 1972; Rescorla, 1971; Solomon et al., 1974; Wagner & Rescorla, 1972; see also Kremer, 1972). This pattern of results has encouraged the view that the latent inhibition effect is essentially an attentional phenomenon—that nonreinforced exposure reduces the ability of a stimulus to command the attention that is necessary for learning, a notion that has been developed theoretically in a variety of ways (e.g., Lubow, 1989; Mackintosh, 1975; Pearce & Hall, 1980; Wagner, 1976, 1981). If a latent inhibitor is a stimulus that does not command this form of attention, it will be difficult to learn about it in any sort of procedure. Thus, it will “pass” a retardation test in both excitatory and inhibitory procedures, but it will show a reduced, or null, ability to disrupt the CR evoked by an excitatory CS in a summation test (i.e., it will not pass a summation test).

In a recent study, we (Liberal et al., 2020) reassessed the role of conditioned inhibition in the latent inhibition phenomenon in terms of an account (Hall & Rodríguez, 2010, 2011) in which inhibitory and attentional processes are seen, not as rivals, but as being jointly responsible for the phenomenon. Specifically, Hall and Rodríguez (2010) assumed that the presentation of a novel stimulus will evoke the expectation that some event will follow; that is, our account assumed the existence of a “stimulus–event” association having some initial strength. During latent inhibition training, as no event follows the presentation of the target stimulus, this initial expectation will be contradicted by experience, and a process parallel to extinction will happen (see also Westbrook & Bouton, 2010). In formal terms, we interpreted extinction in terms of the notion of inhibitory learning adopted by Pearce and Hall (1980; see also Konorski, 1967). Nonreinforced preexposure allows the acquisition of a “stimulus–no-event” association that opposes the effects of the preestablished stimulus–event association (i.e., the expectancy that some event will follow). This inhibitory learning has implications for the attention that will be paid to the stimulus. The attentional principle proposed by Pearce and Hall is that the attention paid to a stimulus (specifically, its associability) is inversely related to its predictive accuracy. The acquisition of the stimulus–no-event association during preexposure will establish the preexposed stimulus as an accurate predictor that nothing will occur, resulting in a reduction in associability.

This account of latent inhibition, in common with any other that uses an attentional construct of this sort, can explain the ability of a preexposed stimulus to pass retardation tests (in both excitatory and inhibitory learning procedures), but it does not predict an effect on a summation test. But, as Liberal et al. (2020) have pointed out, although this is true for the standard latent inhibition training procedure (i.e., repeated nonreinforced exposure to a single stimulus: A, A, A . . .), the Hall and Rodríguez (2010) theory can uniquely predict that for some other preexposure schedules the target stimulus could acquire genuine inhibitory properties that would allow it to pass a summation test. Liberal et al. (2020) present a derivation of this prediction in terms of the formal theory; here, we present a descriptive outline.

According to the Hall and Rodríguez (2010) account, in the standard latent inhibition procedure (i.e., A, A, A . . .) the strength of the inhibitory association (the “A–no-event” association), will come to match and neutralize, but never exceed, the strength of the preexisting excitatory association (the “A–event” association). However, the account anticipates that another form of preexposure—specifically, a preexposure schedule in which the target stimulus, A, is presented in compound with a novel event on each preexposure trial (i.e., exposure to An_1_, An_2_, An_3_ . . .), could turn Stimulus A into a net inhibitor of the occurrence of a subsequent event. The presence of a novel nontarget stimulus (n_1_, n_2_, n_3_ . . .) on each trial will maintain activation of the expectancy that some event will occur, even when the target stimulus has acquired an A–no-event association matching that of the initial, excitatory, A–event association. The strength of the A–no-event association will thus continue to increase, turning A into a net inhibitor of the occurrence of a subsequent event. These net inhibitory properties could be made evident by presenting the preexposed stimulus in compound with a CS that has been separately trained as excitor for some other event and showing that the magnitude of the CR is reduced—that is, a stimulus preexposed under this schedule should be able to pass a summation test.

Liberal et al. (2020) tested this prediction in a series of experiments using an appetitive classical conditioning procedure with rats. In their basic procedure, rats were given extensive exposure to a (visual) target cue (A) and food-reinforced presentations of a different visual cue (X). The summation test consisted of presentations of the AX compound. Some rats were given the standard preexposure procedure (presentations of A alone); others (in the AN condition) received presentations of A in compound with a different novel auditory stimulus (An1, An2, An3 . . . An32) on each preexposure trial. It was found that the presence of A on test produced only a small and nonsignificant reduction ability of X to evoke the CR in subjects given preexposure just to A, but there was a sizeable effect in the AN group given exposure to the varying compounds. Thus, exposure to A, when given along with a range of novel stimuli, enabled it to evidence inhibitory properties on the summation test.

There is nothing in the Hall and Rodríguez account that limits the validity of their predictions to the appetitive conditioning paradigms, and/or to visual and auditory stimuli, such as those used by Liberal et al. (2020). Thus, in the experiments to be reported here we sought to confirm and extend the generality of our previous finding in an aversive conditioning paradigm using flavors as the stimuli. Our experimental design requires a large number of different stimuli (the “n” stimuli) that are readily discriminable by our (rat) subjects. This makes the flavor-aversion-learning paradigm an ideal procedure to employ, as a wide range of different flavors is readily available commercially, and the rat has proven ability to discriminate readily among flavors (e.g., Burn, 2008). In Experiment 1, we first checked that the preexposure schedules with the Target A stimulus (the AN and A-alone preexposure procedures) were capable of producing the basic latent inhibition effect (i.e., a retardation of conditioning). In Experiment 2, we tested whether, as in the study by Liberal et al. (2020), the AN preexposure procedure endowed the Stimulus A with the ability to pass a summation test.

## Experiment 1

This experiment consisted of four groups (see Table 1). All of them received a conditioning trial in which consumption of the target stimulus, Flavor A, preceded an injection of lithium chloride (LiCl), followed by a test trial to assess the level of the aversion acquired by Stimulus A. The groups differed in the treatment that they received during the preexposure phase. Two groups received preexposure to the flavor to be used as the CS: Group A received presentations of the A flavor alone on all the trials of the two preexposure sessions; Group AN received a presentation of A in compound with a different flavored substance on each of the eight trials of the first preexposure session, and this procedure was repeated on the second session. The test performance of these preexposed groups was compared with that of two control groups, not given preexposure to A. Group NP (no preexposure to flavors) received presentations of water during the preexposure phase. A second condition, Group BN, assessed the effects of exposure to the various N stimuli. Subjects in this group received a treatment identical to that received by Group AN, except that nontarget Flavor B was substituted for the Target Flavor A. A latent inhibition effect would be demonstrated if Groups A and AN showed retarded acquisition of an aversion to A, compared with that shown by the control groups.

**Table 1:**
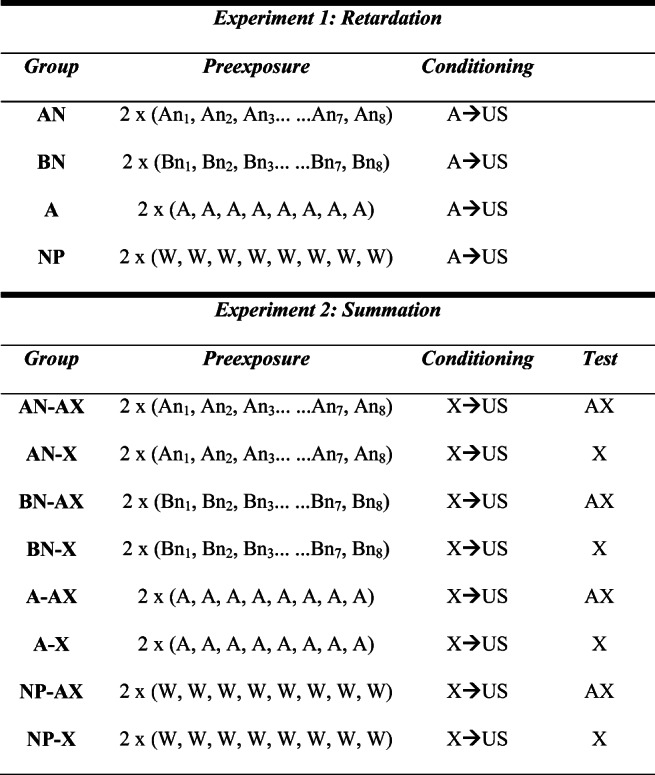
Experimental designs

*Note*. Each letter represents a flavored solution: W = water. There were two sessions of preexposure, each with eight presentations of fluid. The unconditioned stimulus (US) was an intraperitoneal injection of LiCl.

## Method

### Subjects, apparatus, and stimuli

The subjects were 32 male, adult, Sprague Dawley rats (mean ad libitum body weight at the start the start of the experiment: 271 g; range: 233–301 g). They had previously served as subjects in an experiment on appetitive conditioning using operant techniques (with visual and auditory stimuli), but they were naïve to the present stimuli and procedures. In the previous experiment, all the animals experienced a 4-week food-deprivation schedule. When that experiment ended, all the animals returned to ad lib. conditions, and were housed in pairs with continuous access to food and water for a period of 23 days. The next day, the present experiment began; the animals were singly housed in the same colony room as before (artificially lit from 8 a.m. to 8 p.m. each day), with continuous access to food, but restricted access to water (as detailed below).

The solutions used as experimental stimuli were administered in the home cages at room temperature in 50-ml plastic centrifuge tubes, fitted with metal spouts. The following flavored solutions were used. The A and B stimuli, counterbalanced, were a solution of vanilla (2% vol/vol; Le Champion, France) and a solution of almond (2% vol/vol; Le Champion, France). The “n” flavors were: sucrose (2% g/vol), citric acid (2% vol/vol, Merry Sab, Spain), cocoa (2% g/vol; Colacao, Spain), Modena vinegar (2% vol/vol; Borges, Spain), milk (10% vol/vol; Pascual, Spain), quinine (sulphate, 0.005% g/vol), coffee (0.5% g/vol; decaffeinated, Nescafé, Spain), HCl (1% vol/vol). The compound stimuli received by Groups AN and BN during preexposure (e.g., An_1_, An_2_ . . ., An_8_), were mixed so to maintain the abovementioned concentrations of the A (or B) flavor and that of the corresponding “n” flavor. Consumption was measured by weighing the tubes before and after trials, to the nearest 0.1 g. The unconditioned stimulus was an intraperitoneal injection of 0.30 M lithium chloride (LiCl) at 10 ml/kg of body weight.

### Procedure

All procedures relating to the maintenance and use of animals were in accordance with the European Law of Animal Welfare and were approved by the Animal Welfare Committee (CEEA) of the University of the Basque Country (UPV/ EHU).

On the first day of the experiment, the animals were weighed and individually housed; at 18:30 h, the standard bottles of water were removed, and a schedule of water deprivation was initiated. On each of the following 3 days (Days 2, 3, and 4), access to water was restricted to two daily 30-min sessions, starting at 12:00 h and 18:00 h. After the last afternoon session of this phase, the animals were assigned to one of four equal-sized groups (AN, BN, A, and NP, *n* = 8). As the animals had served as subjects in a previous experiment, the group assignment was arranged so that all the conditions of the previous experiment were equally represented in the conditions of the current experiment. For half the animals in each group, the Target Solution A was almond, and the Nontarget Solution B was vanilla, and for the other half the opposite was true.

The preexposure phase consisted of two sessions on Days 5 and 6. On each day, all the animals received eight trials of 5 min, with an interval of 55 min between them. The first trial started at 12:00 h. On each trial, animals were allowed to access to 3 ml of the relevant solution. For animals in Group AN, this solution changed from trial to trial, and consisted of a mixture of the Target Stimulus A and one of the eight “n” flavors (sugar, citric acid, cocoa, vinegar, milk, quinine, decaffeinated coffee, and HCl). The order of presentation of the N solutions within each the session was counterbalanced by assigning to each subject of the group a Latin square condition (i.e., Subject 1: n_1_, n_2_, n_3_, n_4_, n_5_, n_6_, n_7_, n_8_; Subject 2: n_2_, n_3_, n_4_, n_5_, n_6_, n_7_, n_8_, n1; Subject 3: n_3_, n_4_, n_5_, n_6_, n_7_, n_8_, n_1_, n_2_; and so on). The treatment received by animals in Group BN during the two sessions of preexposure was identical to that received by animals in Group AN, except the Target Flavor A was replaced by presentation of the Nontarget Flavor B. Animals in Group A received presentations of the Target Flavor A alone on each of the 16 trials of preexposure. Finally, animals in Group NP received presentations of water on every trial of preexposure.

On the day after completion of preexposure, during the morning session of Day 7 (starting at 12:00), all rats received a conditioning trial in which 10 ml of the Target A solution was presented for 30 min, followed immediately by an injection of LiCl. Free access to water was allowed for 30 min in the afternoon (starting at 18:00). The next day, Day 8, was a recovery day in which all the rats had unrestricted access to water during both morning and afternoon sessions of 30 min. On the morning of Day 9, rats were given a nonreinforced test trial consisting of free access to the Target A solution for 30 min.

### Data analysis

Data from the preexposure and the conditioning trial were examined using the analysis of variance (ANOVA); data from the test trial were examined using analysis of covariance (ANCOVA), with consumption on the conditioning trial as a covariate. Also, where appropriate, *t* tests or Duncan pairwise mean comparison tests were conducted. A criterion of statistical significance of *p* less than .05 was adopted. Effect sizes for the effects from ANOVAs and ANCOVAs are reported as partial eta squared, and those for pairwise comparisons are reported using Cohen’s *d*. The 95% confidence intervals (CIs) around the effect sizes are also reported in brackets following the effect size.

## Results and discussion

Rats did not consume all of the 3 ml offered on each preexposure trial, and the groups differed in this respect. Group mean consumption scores over all preexposure trials were: 1.64 ml (*SEM* = 0.07), 1.68 ml (*SEM* = 0.07), 2.15 ml (*SEM* = 0.08) and 2.14 ml (*SEM* = 0.07) for groups AN, BN, A, and NP, respectively. An ANOVA conducted on these data, with Exposure condition (AN, BN, A, NP) and counterbalancing of the Target A (vanilla or almond) as variables, revealed a significant effect of the exposure condition, *F*(3, 24) = 13.28, *p* < .0001, η_p_^2^ = 0.64, [0.29, 0.73]. Post hoc comparisons with the Duncan test showed that the consumption in groups AN and BN was lower than in Groups A and NP, probably reflecting an effect of neophobia produced by the presence of the substances employed as the N stimuli. This suggestion is supported by the fact that groups AN and BN exhibited particularly low levels of consumption of those compounds containing typically neophobic flavors, such as quinine, acids, vinegar, and coffee. Counterbalancing of A and its interaction with the exposure condition revealed no significant effects, largest *F*(3, 32) = .82, *p* = .494.

**Fig. 1:**
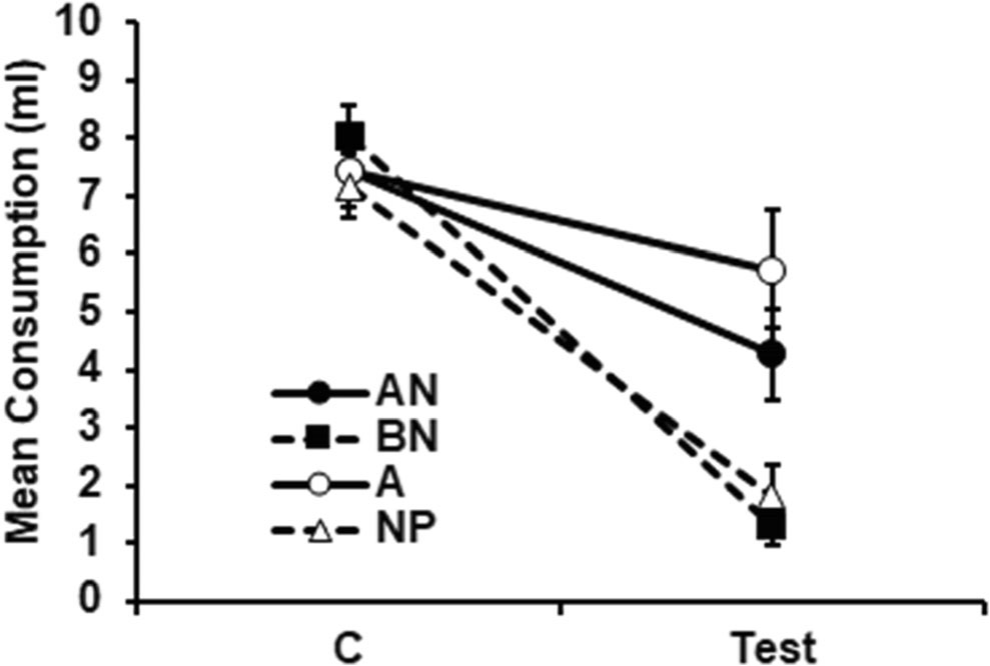
Experiment 1: Mean scores (±*SEM*) for consumption of the A flavor (vanilla or almond, counterbalanced) on the conditioning trial and the subsequent nonreinforced test. Prior to conditioning, Group AN received nonreinforced presentations of A in compound with eight different “n” flavors; Group BN received presentations of a nontarget flavor, B, in compound with the same eight “n” flavors; Group A received presentations to the target flavor A alone; and Group NP received equivalent presentations of water

The results for the conditioning trial and the test are shown in Fig. 1. On the first conditioning trial, all rats drank almost all of the 10 ml made available. An ANOVA conducted on the data for this trial, with the exposure condition (AN, BN, A, NP) and counterbalancing of A as the target (almond or vanilla) as variables, revealed no significant main effects or interaction, largest, *F*(3, 24) = 1.63, *p* = .207. The effect of the conditioning trial, evident on the subsequent test trial, was to suppress consumption in all the groups (i.e., there was evidence of conditioning), but suppression was less marked in the groups preexposed to the target flavor (Groups AN and A) than in the groups for which Flavor A was novel on the conditioning trial (Groups BN and NP). An ANCOVA was conducted on these data, with preexposure condition (AN, BN, A, NP), and counterbalancing of the Target A (vanilla or almond) as factors; consumption during the first conditioning trial was used as a covariate, in order to control for variability potentially caused by different levels of exposure to the CS during that trial. This analysis revealed significant differences among the groups, *F*(3, 23) = 8.4, *p* < .001, η_p_^2^ = 0.54, [0.15, 0.66]. No other effect reached statistical significance, largest, *F*(3, 23) = 1.63, *p* = .21. Post hoc comparisons with the Duncan test showed that on the test trial groups AN and A consumed more than groups BN and NP. Groups AN and A, and groups BN and NP, did not differ from each other in their consumption. The difference between Groups A and NP in test consumption provides a demonstration of the basic latent inhibition effect using a retardation test. Learning was also retarded in the AN group; that the effect was marginally less than that seen in the A group presumably reflects a degree of generalization decrement, with A presented in compound with another flavor being perceived as slightly different from A alone. Presentation of the range of “n” flavors (in compound with Nontarget Flavor B) was without effect on the aversion conditioned to the Target A stimulus.

## Experiment 2

In this experiment, we assessed the ability of the preexposure conditions used in Experiment 1 to allow the Target Stimulus A to pass a summation test. The design of the experiment is shown in Table 1. There were four preexposure conditions, identical to those used in Experiment 1: two groups receiving exposure to A (Groups AN and A), and two groups that received no exposure to A during the preexposure phase (Groups BN and NP). After preexposure, all subjects received aversive conditioning in which a novel CS (Flavor X) was paired with an injection of LiCl. A test was conducted after this conditioning trial. Within each preexposure condition, different subjects were tested with either AX or X. It is possible that, by way of generalization decrement effects, the presence of A will reduce the degree of aversion governed by X in all conditions. But if the treatment given to the AN group has endowed Stimulus A with inhibitory properties (as predicted by the Hall & Rodríguez, 2010, account) we can expect the ability of Stimulus A to interfere with the CR evoked by X should be especially marked in Group AN.

## Method

The subjects were 64 experimentally naïve male Sprague Dawley rats, with a mean ad lib weight of 373 g (range: 314–450 g). Animals were housed and maintained in the same conditions as those described in Experiment 1. The A, B, and N (n1, n2, . . . , n8) stimuli were identical to those used in Experiment 1. A solution of NaCl (1% g/vol) was employed as the X stimulus. As in Experiment 1, the US was an intraperitoneal injection of 0.30 M LiCl, at 10 ml/kg of body weight.

Rats were assigned to one of eight equal-sized groups before the start of preexposure phase: Groups AN–AX, AN–X, BN–AX, BN–X, A–AX, A–X, NP–AX, and NP–X (*n* = 8). The first term of the group labels refers to the treatment received during the preexposure (AN, BN, A, NP), and the second terms refer to the solution with which the group was tested (AX or X). One of the animals in Group AN–AX became ill during the preexposure phase and was removed from the experiment. The procedure in the preexposure phase for the AN, BN, A, and NP conditions was the same as that described in Experiment 1. On the day following the last preexposure session, all animals received a conditioning trial the same as that described in Experiment 1, except that the X stimulus (NaCl) was employed as the CS. The next day was a recovery day in which the rats had unrestricted access to water for 30 min during both the morning and afternoon sessions. The test trial was carried out during the morning session of the following day. On that trial, all the animals received unrestricted access to the appropriate fluid for 30 min. For groups AN–AX, BN–AX, A–AX, and NP–AX, the test solution was the AX compound; for groups AN–X, BN–X, A–X, and NP–X, the test solution was X presented alone. For one rat, in Group NP-X, test consumption could not be assessed because of a leaking bottle. Free access to water was allowed to all the animals during the afternoon session. Details of the procedure not specified here were the same as those described for Experiment 1.

## Results and discussion

The pattern of consumption observed in the preexposure phase was very similar to that observed in Experiment 1—that is, the rats, particularly those given the “n” flavors, did not consume all of the 3 ml offered on each preexposure trial. Group mean daily consumption scores during preexposure were: 1.82 ml (*SEM* = 0.07), 1.87 ml (*SEM* = 0.07), 2.13 ml (*SEM* = 0.07) and 2.27 ml (*SEM* = 0.05) for the preexposure conditions AN, BN, A and NP, respectively. An ANOVA conducted on these data, with the exposure condition (AN, BN, A, and NP) and counterbalancing of the Target A (vanilla or almond) as variables, revealed a significant effect of Exposure condition, *F*(3, 55) = 9.71, *p* < .0001, η_p_^2^ = 0.34, [0.12, 0.47]. Post hoc comparisons with the Duncan test showed that consumption in groups AN and BN was lower than in Groups A and NP. Neither the counterbalancing of the Target A, *F*(1, 55) = 1.54, *p* = .22, nor the interaction between this variable and the exposure condition, *F*(3, 55) = .07, *p* = .974, was significant.

Consumption of the X stimulus on the conditioning trial was similar in the four preexposure conditions. Mean consumption scores on this trial were: 7.70 ml (*SEM* = 0.32), 7.86 ml (*SEM* = 0.40), 8.00 ml (*SEM* = 0.41) and 7.4 ml (*SEM* = 0.54), for conditions AN, BN, A and NP, respectively. An ANOVA conducted on these data, with the preexposure condition (AN, BN, A, NP), and counterbalancing of the Target A (vanilla or almond) as factors, confirmed this impression. This analysis revealed no significant differences among the groups, *F*(3, 55) = 0.28, *p* = .835, no significant effects of counterbalancing, *F*(1, 55) = 1.04, *p* = .312, or of the interaction Exposure Condition × Counterbalancing, *F*(3, 55) = 1.54, *p* = .54.

**Fig. 2:**
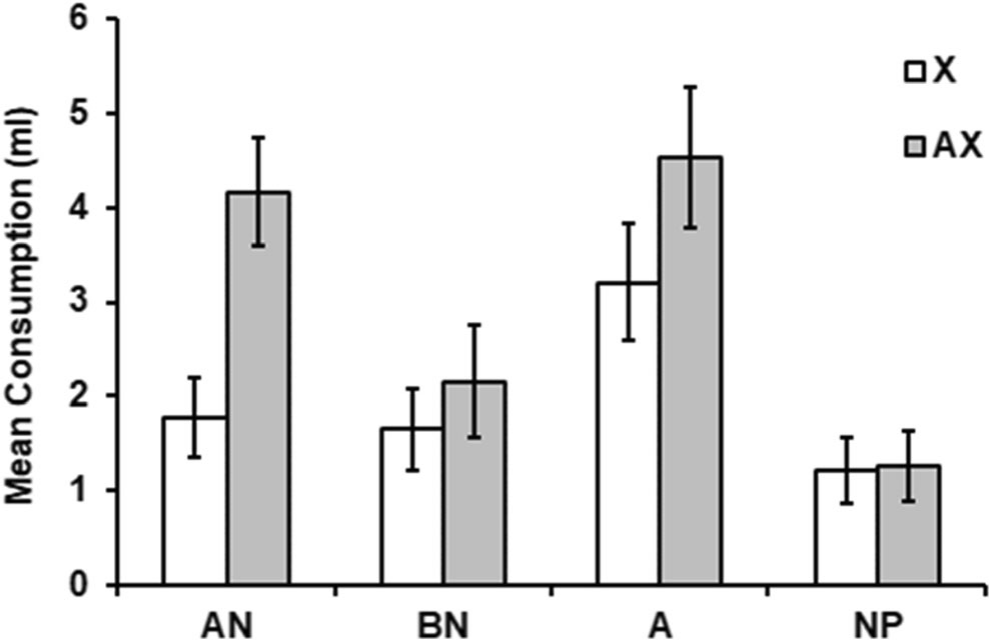
Experiment 2: Mean scores (±*SEM*) for consumption of X and AX flavors (grey and white bars, respectively) on the test trial. Prior to the test all the animals had received one conditioning trial with X as the CS. Prior to this conditioning trial, Group AN received nonreinforced presentations of A in compound with eight different “n” flavors; Group BN received presentations of a nontarget flavor, B, in compound with the same eight “n” flavors; Group A received presentations of the target flavor A alone; and Group NP received equivalent presentations of water

Figure 2 shows group mean consumption during the test, both for the groups tested with the CS (X) alone and those tested with the AX compound. Evidently, conditioning was effective, in that consumption of X was low in all groups tested with X. It was, however, somewhat higher in the A–preexposure condition than in the other three preexposure conditions, AN, BN and NP. This might indicate that generalization of latent inhibition from the preexposure cue to the CS was greater in the A condition, perhaps because there was a higher similarity between the stimulus preexposed in this condition (A) and the CS X than between the stimuli preexposed in the other conditions (the AN and BN compounds) and the CS. Whatever its source, this difference will complicate interpretation of the varying effects of adding A to the CS in the groups tested with AX. As Fig. 2 shows, the effect of adding A was to produce more consumption of AX than X in all preexposure conditions. The difference between X and AX was nominal in the BN and NP conditions, more apparent in the A condition, and especially substantial in the AN condition.

These observations were confirmed by statistical analysis. An ANCOVA was performed on the scores summarized in Fig. 2, with preexposure condition (AN, BN, A, NP), test stimulus (AX or X), and counterbalancing of the A (vanilla or almond) as variables, and with consumption on the conditioning trial as a covariate. As in the previous experiment, this covariate was incorporated in order to control for any variability in the rate of conditioning potentially caused by different levels of exposure to the CS during the conditioning trial. The covariate was significant, *F*(1, 45) = 5.07, *p* = .029, η_p_^2^ = 0.1, [0.00, 0.27]. There were also significant main effects of preexposure condition, *F*(3, 45) = 10.12, *p* < .001, η_p_^2^ = 0.4, [0.15, 0.54], and of test stimulus, *F*(1, 45) = 4.13, *p* = .048, η_p_^2^ = 0.08, [0.00, 0.25], but not of counterbalancing, *F*(1, 45) = 0.10, *p* = .751. Of central theoretical interest, there was a significant Exposure Condition × Stimulus interaction, *F*(3, 45) = 2.87, *p* = .047, η_p_^2^ = 0.16, [0.00, 0.31]. The remaining interactions were not significant, largest, *F*(3, 45) = 2.28, *p* = .09.

Additional analyses performed to explore the source of the Exposure Condition × Stimulus interaction revealed that the preexposure condition modulated the test consumption both of X, *F*(3, 28) = 3.52, *p* = .013, *η*^*2*^_*p*_ = 0.27 (0.00-0.45), and of AX, *F*(3, 26) = 6.71, *p* < .002, η_p_^2^ = 0.43, [0.09, 0.59]. Subsequent comparisons with the Duncan test confirmed that Group A showed more consumption of X than did Groups AN, BN and NP; they also showed that consumption of AX was greater in Groups AN and A than in Groups BN and NP. Critically, the effect of stimulus (i.e., the comparison between the group tested with AX and that tested with X) was significant in the AN preexposure condition, *t*(13) = 3.47, *p* = . 004, *d* = 1.79, [0.55, 2.99], but in none of the other preexposure conditions: largest, *t*(14) = 1.36, *p* = .20.

The comparison performed in the previous literature (e.g., Rescorla, 1969) that has served to establish that a latent inhibitor does not pass a summation test, has usually involved comparing a condition given presentations of the target alone, and a control condition with no exposure. Those conditions are represented in our experimental design by the preexposure conditions A and NP. We conducted an analysis of the test results from these two conditions: an ANCOVA with preexposure condition (A or NP), test stimulus (AX or X), and counterbalancing of A (vanilla or almond) as variables, and the consumption during the conditioning trial as a covariate. The covariate was not significant, *F*(1, 22)= 3.07, *p* = .094. Critically, there was a significant main effect of preexposure condition, *F*(1, 22) = 26.24, *p* < .001, η_p_^2^ = 0.54, [0.22, 0.70], indicating a lower level of response after preexposure to A. No other significant effects were obtained. For the main effect of counterbalancing, *F*(1, 22) = .12, *p* = .732. More critically, neither the main effect of test stimulus, *F*(1, 22) = .21, *p* = .654, nor the Exposure Condition × Stimulus interaction, *F*(1, 22) = 2.66, *p* = .117, was significant. None of the remaining interactions was significant, with the largest *F* value coming from the Counterbalancing × Stimulus interaction, *F*(1, 22) = 3.8, *p* = .064. These results indicate that when comparing the Conditions A and NP, the presence of A did not significantly interfere with the CR elicited by X on test, either when A was novel (group NP) or when it was preexposed on its own (Group A). That is, we replicated the usual result found in the literature in which a standard latent inhibitor fails to pass the summation test.

A similar analysis to that just described but comparing our special condition of latent inhibition training (the AN group) with the control NP group, yields a different pattern of results. An ANCOVA with preexposure condition (AN or NP), test stimulus (AX or X), and counterbalancing of A (vanilla or almond) as variables, and the consumption during the conditioning trial as a covariate, revealed the following results. The covariate was not significant, *F*(1, 21) = 1.81, *p* = .193. The main effect of test stimulus was close to significance, *F*(1, 21) = 4.11, *p* = .055, and there was a significant main effect of preexposure condition, *F*(1, 21) = 14.56, *p* < .001, η_p_^2^ = 0.41, [0.09, 0.61]. Neither the main effect of counterbalancing, *F*(1, 21) = .62, *p* = .441, nor any interaction involving this factor were significant: largest, *F*(1, 21) = 1.57, *p* = .224, for the interaction of Exposure Condition × Counterbalancing. Critically, the Exposure Condition × Stimulus interaction was significant, *F*(1, 21) = 9.76, *p* = .005, η_p_^2^ = 0.31, [0.04, 0.55]. Further analyses performed in order to clarify the source of this interaction showed that groups AN–X and NP–X did not differ in their test consumption, *t*(14) = 1.043, *p* = .315. However, group AN–AX consumed significantly more than group NP–AX, *t*(12) = 4.3, *p* < .01, *d* = 2.15, [0.81, 3.44].

The results analyzed up to this point indicate that Group AN, but not Group A, passes the summation test, as is evidenced by comparison of each of these groups with the standard control group, NP. A further comparison that could prove informative, involves the AN and A groups. Accordingly, we performed an ANCOVA with preexposure condition (AN or A), test stimulus (AX or X), and counterbalancing of A (vanilla or almond) as variables, and the consumption during the conditioning trial as a covariate. This analysis revealed only a significant effect of test stimulus, *F*(1, 22) = 8.08, *p* < .009, η_p_^2^ = 0.26, [0.01, 0.51]. The critical Exposure × Stimulus interaction was not significant, *F*(1, 22) = 1.46, *p* = .23, nor was the covariant *F*(1, 22) = 2.52, *p* = .126, or any of the remaining interactions, with the largest *F* value coming from the Counterbalancing × Stimulus interaction × Exposure condition, *F*(1, 22) = 2.46, *p* = .13. Despite being based on a null result, the absence of the Group X × Exposure interaction in this comparison (AN vs. A) requires us to elaborate our discussion of the overall pattern of results. It indicates that we cannot entirely rule out the possibility that preexposure to A alone can endow that stimulus with the ability to pass the summation test. Perhaps, a smaller effect size in Group A requires greater power to be observed, which would explain why previous studies have failed to detect clear evidence of summation in this condition. This fact, however, does not detract from the main result of the present study, which is to confirm that the exposure condition provided for the AN group was particularly effective in endowing stimulus A with a clear ability to pass a summation test.

In summary, these results indicate that the addition of the Target Stimulus A to a separately trained excitor (X) will reduce the magnitude of the CR when A has been experienced during preexposure along with a variety of novel stimuli. This effect is not seen reliably in any of the control conditions: when A was preexposed alone, when A was novel, or when preexposure involved just the set of novel stimuli in the absence of A. This result accords with the proposal that the treatment given to A in the AN group will endow A with inhibitory properties, allowing it to pass a summation test. This conclusion must be qualified to some extent, given the fact that the CR governed by the test excitor was less in the group given preexposure to A alone than in the other groups. As a consequence, the effects of adding A to X for the A group are assessed against a baseline that is different from that obtaining in the other conditions. It remains the case, however, that, in common with a range of other studies (e.g., Reiss & Wagner, 1972; Rescorla, 1971; Solomon et al., 1974; Wagner & Rescorla, 1972) our study failed to find a significant inhibitory effect in this procedure, but that an effect was obtained for subjects given the AN-preexposure, the procedure that, according to our account should endow A with inhibitory properties.

## General discussion

The present experiments were an attempt to replicate conceptually, in the conditioned flavor aversion procedure, the study by Liberal et al. (2020) which used an appetitive procedure with visual and auditory stimuli. We tested the inhibitory properties of a target stimulus (A) using the double-test (i.e., retardation and summation) criterion (Rescorla, 1971). In the experimental conditions, A was preexposed in the absence of reinforcement either alone (A, A, A . . .) or in compound with a variety of nontarget flavors (An_1_, An_2_, . . . , An_8_). Consistent with previous literature (e.g., Wagner & Rescorla, 1972) and with the results from the study by Liberal et al. (2020), subjects exposed to the target stimulus on its own showed an effect on the retardation test of Experiment 1 (i.e., a latent inhibition effect), but not in the summation test of Experiment 2 (i.e., the presence of A did not interfere effectively with the magnitude of the CR evoked by a separately trained CS). The critical result, however, was that, as in the study by Liberal et al. (2020), subjects preexposed to the target stimulus in compound with a variety of different stimuli showed an effect both on the retardation and the summation tests. These results are predicted by our theoretical account, which proposes that the standard latent inhibition procedure will result in a loss of stimulus associability that will slow further learning, but not influence the effectiveness of a separately trained CS. In contrast, the compound-preexposure procedure used in these experiments is expected to endow the target stimulus not only with a loss of associability but also with inhibitory properties, signaling that no event will follow. The particular circumstances of our compound preexposure procedure (with a novel stimulus added on each trial) were arranged to predict not just a negation of the expectation of a consequent event but also an expectation of no event.

In the original formalization of the Hall and Rodríguez account (2010), it was suggested that the ability of a novel stimulus to activate the expectancy that some consequence will follow might depend on a process of stimulus generalization. The target novel stimulus might be represented as formed of two sorts of features: those that are really novel (A) and those shared with other similar stimuli (S) with which the organism has had experience in the past. Some of these familiar stimuli (BS, CS, DS . . .) would have been followed by some specific event (X, Y, Z . . .), and these various consequences would all be activated, to some extent, by presentation of stimulus A (i.e., AS). Our parsimonious assumption in the 2010 formalization was that the motivational/emotional valence of these outcomes would be multiple, even contrary in some cases. Presentation of A would, therefore, activate representations of different (and sometimes opposing) valence, and thus not producing a clear net activation of a given valence. Because of this, our proposal was that the nature of the representation most strongly activated by a novel stimulus would be just that some event will occur. The results from our study with the appetitive procedure (Liberal et al., 2020) are consistent with this notion, as are those reported here. But the present results are also compatible with a different view.

Consider the results from the preexposure phase, in which we observed reduced levels of consumption in the groups preexposed to the AN and BN compounds. This to be expected given that some of the substances used as “n” flavors (e.g., quinine, coffee, vinegar) are known to produce clear neophobic reactions. One possible interpretation of this sort of response is that the flavor generates an expectation of adverse consequences (of the sort, e.g., produced by poisoning). If so, it is possible that in the case of the flavors, and food in general, a novel stimulus will evoke an expectancy more particular than that supposed by Hall and Rodríguez (2010). It is possible that neophobia-inducing flavors will activate the expectancy that some negative consequence will occur. During exposure to Flavor A alone, that expectancy will be extinguished (e.g., Best, 1975; Kalat & Rozin, 1973), but, during exposure of A along with a range of novel (and neophobic) flavors, A will come to be established as a net inhibitor of a particular consequence such as gastrointestinal upset. This notion raises the interesting possibility that the effect of AN preexposure demonstrated in the present experiments would not be replicated in an appetitive procedure with flavors as target stimuli. If AN preexposure makes A a net inhibitor of the expectancy that some gastrointestinal upset is going to occur, it should not interfere with (and might even facilitate) the expression of an appetitive CR (we note that Liberal et al., 2020, used an appetitive procedure, but with visual and auditory stimuli that are unlikely to generate the neophobic response of relevance here).

Leaving this speculation aside we return to the central conclusions prompted by the present results. Using the taste-aversion procedure, we have identified a preexposure schedule that will endow the preexposed stimulus with the ability to pass both retardation and summation tests. We have thus replicated the central finding of the study by Liberal et al. (2020), which used an appetitive procedure with auditory and visual cues. Taken together, these results give support to an interpretation of the effects of nonreinforced preexposure effects that gives an important role to inhibitory learning. Further research will be needed to clarify the nature and the valence of the central representation of the potential consequences of an event, the representation that is taken to be subject to inhibition.

Finally, we may note possible translational contributions from our findings to the field of fear learning and the design of treatments for anxiety disorders. Several current interventions involve providing patients with training conditions intended to produce conditioned inhibitors able to interfere with CRs of anxiety/fear (e.g., Lebois et al., 2019). A negative aspect of this sort of training is that it involves giving patients the unpleasant experience of exposure to CSs or USs that evoke negative emotional responses. But our results indicate that, by presenting it in compound with a range of new stimuli (n_1_, n_2_, n_3_ . . .), it is possible to endow an initially neutral stimulus (A, in our experiments) with the ability to inhibit expectation of a consequence. Thus, we could have found training conditions able to generate conditioned inhibitors of aversive stimuli, but without need to expose subjects to the damaging effects of such stimuli.
